# Probing of heavy metals in the feathers of shorebirds of Central Asian Flyway wintering grounds

**DOI:** 10.1038/s41598-020-79029-z

**Published:** 2020-12-17

**Authors:** Jeganathan Pandiyan, Rajendran Jagadheesan, Ganesan Karthikeyan, Shahid Mahboob, Khalid A. Al-Ghanim, Fahad Al-Misned, Zubair Ahmed, Kaliyamoorthy Krishnappa, Kuppusamy Elumalai, Marimuthu Govindarajan

**Affiliations:** 1 Department of Zoology and Wildlife Biology, A.V.C. College, Mannampandal, Mayiladuthurai, Tamil Nadu 609 305 India; 2 Department of Zoology, College of Science, King Saud University, Riyadh, 11451 Saudi Arabia; 3 Department of Advanced Zoology & Biotechnology, Government Arts College for Men (Autonomous), Chennai, Tamil Nadu 600035 India; 4 Unit of Vector Control, Phytochemistry and Nanotechnology, Department of Zoology, Annamalai University, Annamalainagar, Tamil Nadu 608 002 India; 5 Unit of Natural Products and Nanotechnology, Department of Zoology, Government College for Women (Autonomous), Kumbakonam, Tamil Nadu 612 001 India

**Keywords:** Environmental impact, Ecology, Environmental sciences

## Abstract

The study is intended to deliver the incidence of heavy metals in the feathers of shorebirds from two important Central Asian Flyway (CAF) migratory shorebirds wintering sites such as the Point Calimere Wildlife Sanctuary (PWLS) and Pichavaram Mangrove Forest (PMF), India. Feathers of fifteen species of shorebirds and seven different metals viz., Cu, Cr, Co, Pb, Hg, Ni and Zn were analyzed. Zn was highest in Dunlin, Little-ringed Plover, Marsh Sandpiper, and Common sandpiper, Ni showed highest in Little ringed plover, and Common sandpiper, Co, Cr, and Cu were maximum in Little stint, Marsh sandpiper, and Dunlin, respectively. The Hg was higher in Black-winged stilt, Common redshank, Curlew Sandpiper, Eurasian curlew, Lesser Sand-plover, Temminck’s stint, Kentish plover, Spotted redshank, and Wood sandpiper, the Pb found highest in Kentish plover, Painted stork, Spotted redshank, Wood sandpiper, Eurasian Curlew, and Lesser sand-plover. The concentration of metals showed significant variations among the species of shorebirds studied (P < 0.001). The mercury negatively correlated with the other metals than the other six metals studied in both the wetlands. The order of metal concentration in the feathers of shorebirds was Zn > Ni > Co > Cr > Cu > Pb > Hg. Nevertheless, the current study revealed that the level of metals in the shorebirds is alarming; since the PWLS and PMF are located along the CAF routes, it needs intensive studies on various pollutions to manage both the resident as well as migratory shorebirds.

## Introduction

Shorebirds are traveling thousands of kilometers annually to fulfill their energetic demands^1^. Several shorebirds migrate annually from their breeding grounds to wintering grounds to meet out their energetic demands. In India, several wintering grounds support many shorebirds species during their migration, especially in India's southern parts, for example, Point Calimere Wildlife Sanctuary (PWLS), Kodikkarai and Pichavaram Mangrove Forests (PMF), Tamil Nadu, India. These wintering grounds act as important stopover sites and as the CAF and EAAF migratory routes for several shorebird species during their migration^2–8^. However, of late, these wetlands have been polluted by various developmental processes, applying various fertilizers and pesticides in farmlands, and undertaking intensive anthropogenic practices^9,10^. Among the pollutants, the most prevalent are heavy metals.

In wildlife, aquatic organisms are under severe threat due to pollution, especially metals, in which the tertiary consumer is affected adversely being at the top of the trophic structure, especially shorebirds. Heavy metals have highly contaminated the environment posing severe risks to humans and wildlife^11,12^. In addition to that, the loss of coastal biodiversity, including shorebirds, are occurred beyond any reasonable doubt^13^. These losses happen so intensely for the past 50 years, positively correlated with the global economic growth^14–16^. Heavy metal accumulation could influence the shorebird physiology, adversely affecting their feeding habits, growth, age, reproductive stage, moulting, migration and distribution^17–19^.

Studies have reported that over the past twenty years, emerging of factories and unindustrialized farming activities in several regions affected with severe environmental pressures and threats for various properties of soil and sediments^20^, aquatic ecosystems^21^, fish^22^, various tissues in birds^23–25^. Naturally, heavy metals are not biodegradable elements in an ecosystem; the metal could magnify in the superior levels of food chains through food and feeding preferences of several organisms^26^. Heavy metals are major ecological toxic pollutants that affect the food and feeding behaviour and evolutionary aspects of various species^27,28^.

The current study planned to analyze heavy metal concentration through non-invasive methods, particularly by using the shorebirds' feathers. Several studies examined the level of heavy metals found at various tissues and organs of various species^29^, feathers^30^, eggs^31^, blood^32^, eggshells^33^, and liver and kidney and prey samples of migrant shorebirds^30^. Since common and proper ethical attentions do not permit the free-ranging animals' sacrifice for biomonitoring^34^, non-destructive bio-monitoring tools such as analysis of feathers, eggs, and food and prey items avian communities have been used as bioindicators for the evaluation of pollution, particularly metals^26^.

Among the metals, the current study focussed only on seven metals for analysis as studies shows that metals such as chromium (Cr), copper (Cu), cobalt (Co), mercury (Hg), lead (Hg), nickel (Ni) and zinc (Zn) enter the food chain. They can bio-magnification process^31,35^ and the metals are highly toxic, which will affect the plant and animals' life in an ecosystem^36^. Besides, a study revealed that more significant accumulation of these metals extensively threatens the breeding ecology of various wildbirds^35^.

Several studies piloted the analysis of heavy metals from various sources of aquatic and terrestrial organisms, but the occurrence of metal contamination in feathers of shorebirds, including migratory shorebirds from the two wintering grounds, i.e., PWLS and PMF, Tamil Nadu, India, not inferred. Besides, the classical witness, i.e., the Spoonbill Sandpiper (*Calidris pygmaea*), an endangered shorebird, was recorded at the Point Calimere Wildlife Sanctuary in 1980^37^, 1985^38^ and 2004^39^ but has not been seen since then due to the degradation of the habitat and other ecological factors (not confirmed). A recent study reported that the density, diversity and species richness of many migratory shorebirds have drastically declined at the wintering grounds^40^. Studies on such aspects are also negligible at the study sites. A study by reported that halophytic plants at the Pichavaram Mangrove have a higher concentration of metals than mangrove speciesy^9^. Nevertheless, no study was conducted so far on the feathers of shorebirds for the presence of metals at these two wintering grounds. Hence, the current study's main aim is to evaluate the level of heavy metal in different species of shorebirds to get first-hand information at these two wintering grounds.

## Study area

### The Point Calimere Wildlife Sanctuary

**Figure 1:**
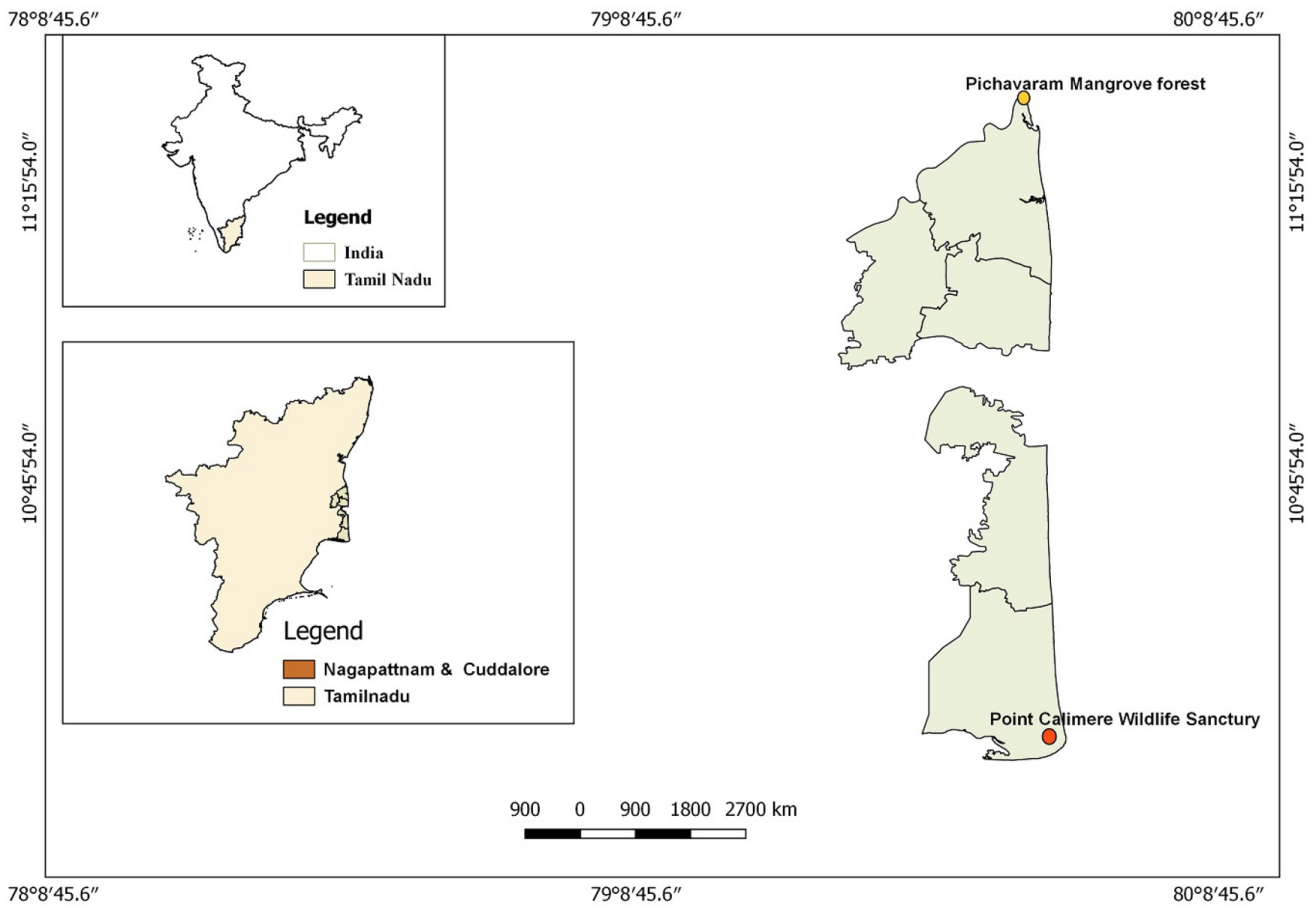
Map showing the Point Calimere Wildlife Sanctuary, Kodikkarai, Pichavaram Mangrove Forest, Cuddalore District, Tamil Nadu, India. The map is generated by using ArcGIS 10.0; ENVI + IDL License No.239509; The software is purchased by the Department of Zoology and Wildlife Biology, AVC College (Autonomous),Mannampandal, Mayiladuthurai, Tamil Nadu, India; The ArcGIS webpage: https://www.arcgis.com/index.html.

The Point Calimere Wildlife Sanctuary (PWLS) is one of the Ramsar sites in India, situated 10°18″ N, 79°51″ E, Great Vedarnyam Swamp, Kodikkarai, Tamil Nadu, India (Fig. 1). The water source for the PWLS is obtained through rainwater during the monsoon and but the sanctuary is gradually dried during summer and ultimately, the entire swamp will become a small pool of water in peak summer. The swamp area, which is the important foraging site for shorebirds in PWLS, it is separated from the Bay of Bengal and Palk Strait by a narrow stretch of sandbanks with several channels such as “Manavaykal” and “Sellakkani” sea mouths. The swamp area is consisting of a diverse aquatic ecosystem such as both fresh and seawater. In the swamp, two industrial salt units are producing edible and industrial salts. The sanctuary climate is monsoonal, and the significant rainfall occurs in north-east monsoon from October to December, with the maximum rainfall of about 1000–1500 mm. The sanctuary's maximum temperature was 34 °C, which is observed for May and the minimum was 22 °C in December. Annually the swamp habitat of PWLS shows strong winds during June and July. Several resident migratory shorebirds use this sanctuary as a feeding ground and few species use breeding ground^3,37,41^.

### Pichavaram Mangrove Forest (PMF)

The Pichavaram Mangrove Forest (PMF) is located at 11° 29′ N: 79^o^46′ E, the east coast of Tamil Nadu, India (Fig. 1). The PMF is covering 1100 hectares, consisting of 51 islets, a natural mangrove forest with the Vellar-Coleroon estuarine complex^2,42^. In the total area, 50 percent is forest, 40 percent waterways and the rest of the 10 percent is sand and mudflats in which *Avecenia* and *Rhisophora* are major mangrove species^43^. It is incredibly productive and yields nearly eight tonnes of organic plant waste/ha/year^44^. The PMF is attracting several species of migratory shorebirds annually^3^. The physical and chemical factors influence the density and their foraging behaviour water birds in PMF^45^. The rare and near-threatened shorebirds visited in PMF annually^5^. The PMF threatens the migratory and resident migratory shorebirds since it is polluted and degraded through various actions^10^.

## Methods

### Collection and digestion of bird feathers

Fifteen species of shorebirds such as Black-winged Stilt (*Himantopus himantopus*), Common redshank (*Tringa tetanus*), Common Sandpiper (*Actitis hypoleucos*), Curlew Sandpiper (*Calidris ferruginea*), Dunlin (*Calidris alpina*), Eurasian Curlew (*Numenius arquata*), Kentish Plover (*Charadrius alexandrines*), Lesser Sandplover (*Charadrius mongolus*), Little-ringed plover (*Charadrius dubius*), Little stint (*Calidris minuta*), Marsh sandpiper (*Tringa stagnatilis*), Painted Stork (*Mycteria leucocephala*), Spotted Redshank (*Tringa erythropus*), Temminck's Stint (*Calidris temminckii*) and Wood Sandpiper (*Tringa glareola*) were used for the metal study, in which 12 dead carcasses of shorebird species from PWLS (Ramsar site) and 13 dead carcasses of shorebird species from PMF were collected and the other species such as Temminck's Stint in 2015, Dunlin in 2016 and Little stint in 2017 from PWLS and Black winged stilt in 2016 and Dunlin in 2017 from the PMF were caught using mist net and the shorebirds were released into the field without any harming the birds after collecting their breast feathers for analysis. Metal analysis, the breast feathers were preferred since they represent the plumage and are less influenced by the molt than flight feathers^46^. Three individuals of each species were used and the feathers were cleaned in double-distilled H_2_O to remove outwardly absorbed heavy metals. The washed feathers were dehydrated for about 24 h in a hot air oven at 60 °C till reaching constant dry mass and weighed to the nearest 0.001 g^47^. The feathers were also processed with 65% nitrogenous acid and 70% HClO_4_ (4:1 ratio) for proper digestion of the feather for metal analysis^48^. The digested feather samples were diluted by adding 10 ml of deionized H_2_O and the samples were kept in (C_3_H_6_)_n_ metal-free flasks at 20 °C till analysis of metal in AAS^49^.

### Quality control (QC)

The quality control (QC) sample was injected for every ten samples to obtained proper readings. Nevertheless, the variations of QC in metal concentrations were more significant than 15 percent. Furthermore, to avoid samples' impurity, the reagent blanks were included for each batch of metal sample analysis. Average values were obtained from six replicate for a proper determination of metals. The accuracy of the analytical measurement was expressed as relative standard deviation (RSD) ranging from five percent to ten percent and it was derived from the ${\text{SD}}/\overline{{\text{x}}}$. Separate calibration curves were prepared for each metal at different concentrations, i.e., 0.5, 1, 2, 5 and 10 ppm of standard solutions. In addition to that, the daily working solutions were prepared through proper dilutions of standard stock solution with the combination of 65% (v/v) HNO_3_, 30% (v/v) H_2_O_2_ and H_2_O (v/v/v = 1:1:3). The reagent blank was used to set the instrument with ‘0’ concentration for every sample. All glassware was cleaned with deionized H_2_O before use, soaked in 30 percent HNO_3_ overnight, rinsed in deionized water, and air-dried. The analyses were performed by using double beam AAS and the results were described as ppm^50^.

### Statistical analysis

The results are described as mean ± standard error. The Shapiro–Wilk’s normality test was applied before employ statistical tests. Analysis of variance (ANOVA) was applied to understand the variations of heavy metals among shorebirds. The inter-correlation analyses have applied to explore the relationships among the seven metals of the shorebirds examined. Statistical analyses were executed using SPSS 16.0. Results of the data were inferred using typical statistical procedures^51^.

## Results

The study was conducted from 2014 to 2019 and seven different heavy metals viz., chromium (Cr), cobalt (Co), copper (Cu), lead (Pb), mercury (Hg), nickel (Ni), and zinc (Zn) were examined from the feathers of the shorebirds viz., Black-winged Stilt, Common redshank, Common sandpiper, Curlew Sandpiper, Dunlin, Eurasian Curlew. Kentish Plover, Lesser Sandplover, Little-ringed plover, Little stint, Marsh sandpiper, Painted Stork, Spotted Redshank, Temminck's Stint and Wood Sandpiper, at PWLS, Kodikkarai (Ramsar Site), and PMF, India. Among the fifteen species, the three species, such as Eurasian Curlew, Curlew Sandpiper and Painted stork, are globally characterized as Near Threatened (IUCN, 2020).

The zinc (Zn) was highest in Dunlin (90.08 ± 4.29 ppm), Little ringed plover (75.5 ± 2.25 ppm), Little stint (419.09 ± 178.1 ppm) and Marsh sandpiper (91.9 ± 1.42 ppm). The nickel (Ni) was highest in Common sandpiper (70.9 ± 9.75 ppm), the lead (Pb) was more meaningful content in Black-winged stilt, Common Redshank, Curlew sandpiper, Eurasian Curlew, Lesser sand plover and Temminck’s stint (9.6 ± 0.42, 9.6 ± 0.26, 9.6 ± 0.57, 6.9 ± 1.09, 9.5 ± 0.52 and 6.1 ± 0.34 ppm), respectively. The mercury (Hg) was highest in Kentish plover (8.3 ± 1.03 ppm), Painted stork (8.1 ± 0.64 ppm), Spotted redshank (7.8 ± 0.55 ppm) and Wood sandpiper (7.7 ± 0.82 ppm) at PWLS. On the other hand, Zn was highest in Common sandpiper (95.6 ± 4.08 ppm) and Little ringed plover (328.4 ± 140.21 ppm). Copper (Cu), Chromium (Cr) and Cobalt (Co) were highest in Dunlin (87.2 ± 10.62 ppm), Marsh Sandpiper (114.7 ± 5.39 ppm) and Little stint (128.7 ± 54.72 ppm), respectively. The lead (Pb) was highest in black-winged stilt (10.2 ± 1.20 ppm), Common redshank (10.6 ± 1.53 ppm), Curlew sandpiper (12.005 ± 0.92 ppm), Temminck’s stint (10.6 ± 0.58 ppm), Kentish plover (8.3 ± 0.66 ppm), Spotted redshank (10.6 ± 0.58 ppm) and Wood sandpiper (9.5 ± 0.39 ppm). Mercury (Hg) was highest in Eurasian Curlew (7.7 ± 0.46 ppm), Lesser sand plover (5.8 ± 0.79 ppm) and Painted stork (8.6 ± 0.60 ppm), at PMF (Tables 1, 2).

**Table 1:** Different heavy metal concentration recorded from the feathers of the different species of shorebird in the Point Calimere Wildlife Sanctuary, Kodikkarai, Tamil Nadu, India.

Shorebirds	Metals (ppm)						
	Mercury (Hg)	Copper (Cu)	Chromium (Cr)	Cobalt (Co)	Lead (pb)	Nickel (Ni)	Zinc (Zn)
Black winged Stilts	6.1 ± 0.43	0.4 ± 0.25	1.595 ± 0.20	0.7 ± 0.13	9.6 ± 0.42	0.6 ± 0.024	2.559 ± 0.35
Common red shank	6.01 ± 0.50	0.7 ± 0.05	1.7 ± 0.57	0.6 ± 0.12	9.6 ± 0.26	0.6 ± 0.16	2.041 ± 0.29
Common sandpiper	0.2 ± 0.05	14.1 ± 2.51	1.7 ± 0.09	8.4 ± 0.29	0.002 ± 0.001	70.9 ± 9.75	64.2 ± 3.79
Curlew Sandpiper	5.4 ± 0.26	0.5 ± 0.23	0.6 ± 0.04	0.5 ± 0.14	9.6 ± 0.57	0.4 ± 0.27	2.03 ± 0.29
Dunlin	0.5 ± 0.07	20.9 ± 2.72	2.6 ± 0.07	2.6 ± 0.17	2.8 ± 0.03	19.05 ± 0.41	90.08 ± 4.29
Eurasian Curlew	5.3 ± 0.67	0.5 ± 0.11	1.4 ± 0.17	0.5 ± 0.05	6.9 ± 1.09	0.5 ± 0.22	1.4 ± 0.18
Kentish Plover	8.3 ± 1.03	0.2 ± 0.21	0.7 ± 0.25	0.8 ± 0.53	6.06 ± 0.94	0.7 ± 0.47	1.5 ± 0.01
Lesser Sand plover	7.7 ± 1.05	0.3 ± 0.15	0.5 ± 0.17	0.6 ± 0.08	9.5 ± 0.52	0.6 ± 0.15	2.5 ± 0.27
Little ringed plover	5.2 ± 1.97	16.4 ± 0.60	2.7 ± 0.22	0.6 ± 0.02	19.5 ± 9.76	114.8 ± 1.18	75.5 ± 2.25
Little stint	6.07 ± 0.33	71.4 ± 0.83	28.01 ± 0.94	9.7 ± 4.40	28.5 ± 2.31	15.2 ± 1.82	419.09 ± 178.1
Marsh sandpiper	0.05 ± 0.002	82.3 ± 2.32	22.1 ± 0.31	53.08 ± 0.94	35.5 ± 1.18	81.9 ± 35.96	91.9 ± 1.42
Painted Stork	8.1 ± 0.64	0.6 ± 0.11	2.7 ± 0.65	0.5 ± 0.06	2.4 ± 0.30	1.3 ± 0.75	1.5 ± 0.12
Spotted Red shank	7.8 ± 0.55	0.6 ± 0.09	2.6 ± 0.61	0.3 ± 0.16	5.4 ± 0.73	0.4 ± 0.20	2.1 ± 0.20
Temminck's Stint	5.01 ± 0.49	0.6 ± 0.15	1.3 ± 0.11	0.7 ± 0.14	6.1 ± 0.34	0.4 ± 0.22	1.7 ± 0.09
Wood Sandpiper	7.7 ± 0.82	1.3 ± 0.27	2.9 ± 1.03	1.03 ± 0.65	3.1 ± 0.26	1.4 ± 0.52	1.7 ± 0.51

Values are Mean ± SE [ppm].

**Table 2:** Different heavy metal concentration recorded from the feathers of the different shorebird species in Pichavaram Mangrove Forest, Pichavaram, Cuddalore District, Tamil Nadu, India, (Values are Mean ± SE [ppm]).

Shorebirds	Metals (ppm)						
	Mercury (Hg)	Copper (Cu)	Chromium (Cr)	Cobalt (Co)	Lead (Pb)	Nickel (Ni)	Zinc (Zn)
Black winged Stilts	5.2 ± 0.23	0.3 ± 0.16	0.3 ± 0.02	0.6 ± 0.07	10.2 ± 1.20	0.6 ± 0.18	3.6 ± 0.35
Common red shank	5.6 ± 0.59	0.3 ± 0.32	1.3 ± 0.28	0.8 ± 0.49	10.6 ± 1.53	0.3 ± 0.18	0.6 ± 0.22
Common sandpiper	5.4 ± 0.27	14.9 ± 1.4	4.1 ± 0.48	0.05 ± 0.05	1.03 ± 0.51	7.3 ± 5.81	95.6 ± 4.08
Curlew Sandpiper	5.5 ± 0.44	0.5 ± 0.21	0.2 ± 0.12	0.5 ± 0.14	12.005 ± 0.92	2.4 ± 0.31	1.6 ± 0.47
Dunlin	0.9 ± 0.03	87.2 ± 10.62	0	30.8 ± 16.52	2.9 ± 1.63	1.4 ± 0.32	18.3 ± 1.96
Eurasian Curlew	7.7 ± 0.46	0.2 ± 0.04	2.5 ± 0.34	0.5 ± 0.04	6.3 ± 1.04	0.3 ± 0.16	1.5 ± 0.17
Kentish Plover	7.2 ± 0.68	0.5 ± 0.09	1.8 ± 0.06	0.6 ± 0.04	8.3 ± 0.66	0.5 ± 0.21	1.5 ± 0.18
Lesser Sand plover	5.8 ± 0.79	0.5 ± 0.13	0.5 ± 0.17	0.4 ± 0.15	4.9 ± 0.36	0.3 ± 0.08	1.8 ± 0.08
Little ringed plover	8.3 ± 0.33	12.2 ± 0.39	2.9 ± 0.33	4.4 ± 0.54	1.06 ± 0.53	29.6 ± 13.38	328.4 ± 140.21
Little stint	0.1 ± 0.08	15.6 ± 0.53	88.85 ± 5.97	128.7 ± 54.72	14.8 ± 1.29	31.6 ± 0.54	46.2 ± 1.85
Marsh sandpiper	0.2 ± 0.01	32.3 ± 6.69	114.7 ± 5.39	30.5 ± 1.53	23.1 ± 1.33	61.3 ± 3.36	48.2 ± 0.75
Painted Stork	8.6 ± 0.60	0.3 ± 0.26	1.4 ± 0.26	0.7 ± 0.07	8.1 ± 0.85	0.6 ± 0.11	1.6 ± 0.17
Spotted Red shank	7.5 ± 0.35	1.9 ± 0.42	1.5 ± 0.15	0.9 ± 0.19	10.6 ± 0.58	0.5 ± 0.18	3.06 ± 0.62
Temminck's Stint	3.9 ± 0.61	0.4 ± 0.33	1.8 ± 0.14	0.5 ± 0.05	11.9 ± 1.40	0.4 ± 0.25	1.04 ± 0.16
Wood Sandpiper	5.1 ± 0.29	0.3 ± 0.20	1.8 ± 0.25	0.6 ± 0.14	9.5 ± 0.39	0.8 ± 0.36	1.8 ± 0.18

In feathers, the Cu was negatively correlated with the mercury in both the wetlands. The Cr showed a positive correlation with Cu (r = 0.944) at PWLS and negatively correlated with mercury (r = − 0.686) at PMF. The Co was positively correlated with Cu (r = 0.812) and chromium (r = 0.688) and negatively correlated with mercury (r = − 0.550) and r = − 0.561) at PWLS and PMF respectively. The Pb positively correlated with Cu (r = 0.804), Cr (r = 0.786) and Co (r = 0.672) at PWLS. However, in the PMF the lead correlated positively with Cr and Co and negatively with Hg (r = − 0.483). The Ni also positively correlated with Cu (r = 0.461), Co (r = 0.478) and Pb (r = 0.431) on the other hand the Ni showed positive correlation with Cr (r = 0.461), cobalt (r = 0.478) and Hg (r = 0.431) at PMF. The Zi have positive correlation with the Cu (r = 0.659), Cr (r = 0.700) and Hg (r = 0.476). Nevertheless, at the PMF, the Zi showed a negative correlation with the Hg (r = − 0.322) (Table 3). Nevertheless, the Hg not positively correlated with any metals studied in shorebirds of both the wetlands.

**Table 3:** Inter-correlational analysis of metals evaluated from the feathers of shorebirds, Point Calimere Wildlife Sanctuary and Pichavaram Mangrove Forest (Bold letter values are indicates significant differences at 0.05).

	Pb	Ca	Cu	Cr	Co	Hg	Ni	Zn
**Point Calimere Wildlife Sanctuary**								
Pb	1							
Cd	0.178	1						
Cu	− **0.478**	0.012	1					
Cr	− 0.255	− 0.009	**0.944**	1				
Co	− **0.550**	− 0.194	**0.812**	**0.688**	1			
Hg	− 0.156	− 0.006	**0.804**	**0.786**	**0.672**	1		
Ni	− **0.509**	0.179	**0.461**	0.269	**0.478**	**0.431**	1	
Zi	− 0.175	0.149	**0.659**	**0.700**	0.201	**0.476**	0.180	1
**Pichavaram Mangrove Forest**								
Pb	1							
Cd	0.324	1						
Cu	− **0.593**	0.047	1					
Cr	− **0.686**	− 0.154	0.235	1				
Co	− **0.561**	− 0.079	0.268	**0.570**	1			
Hg	− **0.483**	− **0.440**	− 0.096	**0.709**	**0.352**	1		
Ni	− **0.480**	0.009	0.256	**0.848**	**0.415**	**0.505**	1	
Zi	0.146	0.674	0.092	0.053	0.037	− **0.322**	0.278	1

The accumulation level of heavy metals in the shorebirds is as follows: zinc > nickel > cobalt > chromium > copper > lead > mercury. The concentration of metals showed significant variations among the shorebirds studied (p < 0.001).

## Discussion

Heavy metals naturally exist in any environment, which does not undergo biodegradation in the habitats where they are released, and hence undergo bio-magnification in the exposed organisms. Habitats such as mudflats, sand flats and other coastal wetlands are frequently used by the shorebirds as wintering stopover sites to refuel their energetic demands. These habitats are not exceptions, which also get contaminated by heavy metals from various sources. Globally, wintering grounds are considered high-level priority habitats like wetlands and are declared as Important Bird Areas, Ramsar sites, International Single Species Action Plan habitats, etc. The PWLS has been upgraded into one of the Ramsar sites in 2002. The PMF is also one of the important stopover sites for migratory birds during migration^3,45,52^. These habitats need assessment concerning their quality, particularly the evaluation of heavy metals viz., Cu, Cr, Co, Pb, Hg, Ni and Zn and the status of other pollutants organochlorine and organophosphorus pesticides etc., and these habitats attract various species annually, including ‘EN’ and ‘NT’ species of shorebirds. Heavy metals are considered “vital ecological importance since they cannot be totally degraded, or destroyed during the self-purification process of water; rather, they accumulate in the reservoirs and enter the food chain from there”^53^. Hence, the present investigation assumes more vital since it is the first of its kind in these habitats. The study brings out that the accumulation pattern of heavy metals in the feathers of shorebirds is as follows: zinc > nickel > cobalt > chromium > copper > lead > mercury. The current results revealed that metals' level showed significant differences among the shorebirds (P < 0.001). The accumulation of metals in the fifteen species shorebirds perhaps not only from both the wetlands but also they might have consumed the metals from various stopover sites during their migration.

**Figure 2:**
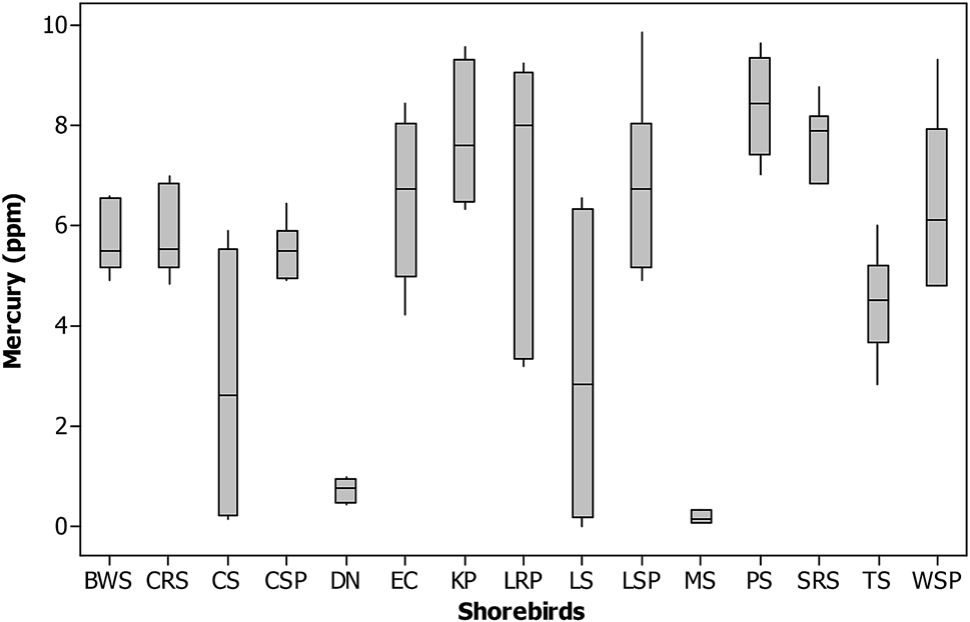
Box plot showing the concentration of mercury (ppm) recorded in different shorebird species at Point Calimere Wildlie Sanctuary, Kodikkarai and Pichavaram Mangrove Forest, Pichavaram, east coast of Tamil Nadu, India. (Shorebirds: *BWS* black winged Stilt; *CRS* common red shank; *CS* common sandpiper; *CSP* curlew Sandpiper; *DN* Dunlin; *EC* Eurasian Curlew; *KP* kentish plover; *LRP* little ringed plover; *LS* little stint; *LSP* lesser sand plover; *MS* marsh sandpiper; *PS* painted stork; *SRS* spotted redshank; TS = Temminc’s stint; *WSP* wood sandpiper).

**Figure 3:**
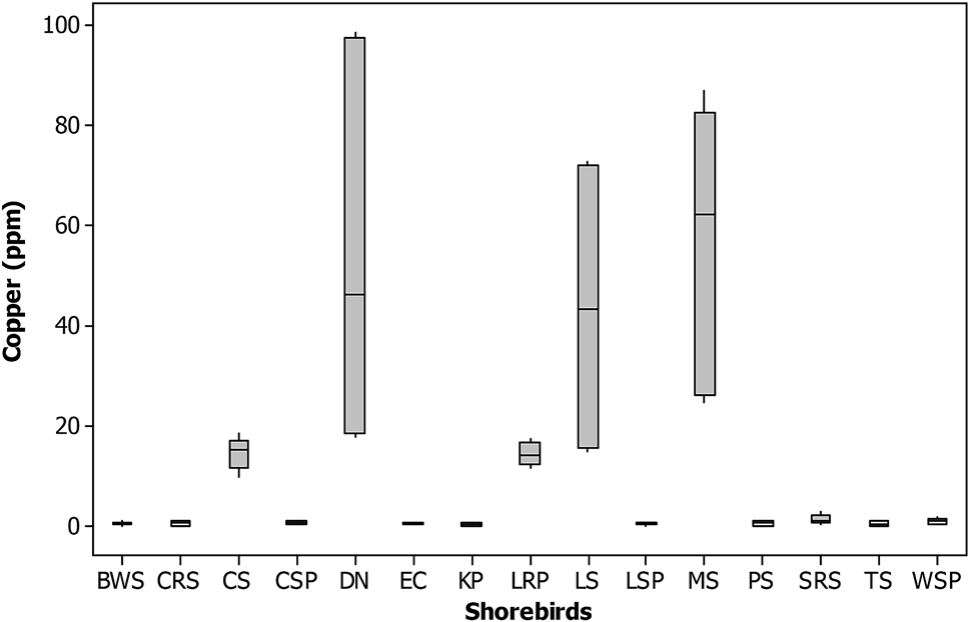
Box plot showing the concentration of copper (ppm) recorded in different shorebird species at Point Calimere Wildlie Sanctuary, Kodikkarai and Pichavaram Mangrove Forest, Pichavaram, east coast of Tamil Nadu, India.

**Figure 4:**
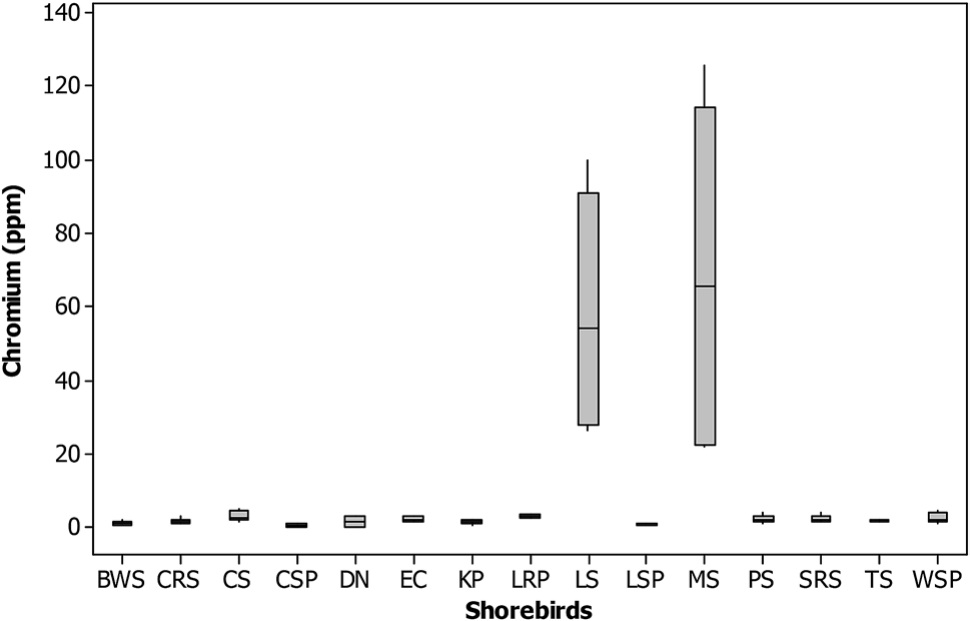
Box plot showing the concentration of chromium (ppm) recorded in different shorebird species at Point Calimere Wildlie Sanctuary, Kodikkarai and Pichavaram Mangrove Forest, Pichavaram, east coast of Tamil Nadu, India.

**Figure 5:**
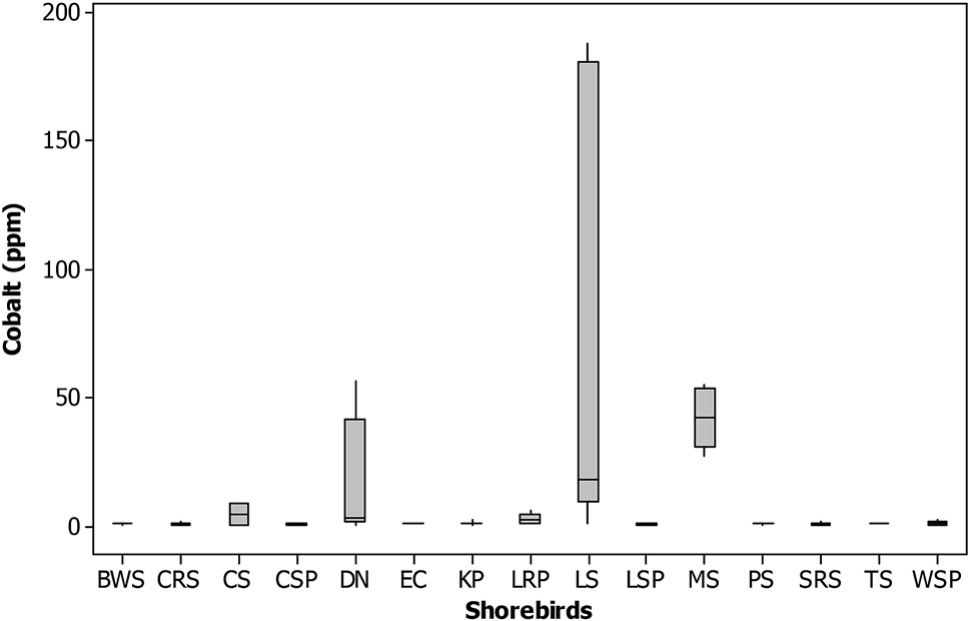
Box plot showing the concentration of cobalt (ppm) recorded in different shorebird species at Point Calimere Wildlie Sanctuary, Kodikkarai and Pichavaram Mangrove Forest, Pichavaram, east coast of Tamil Nadu, India.

**Figure 6:**
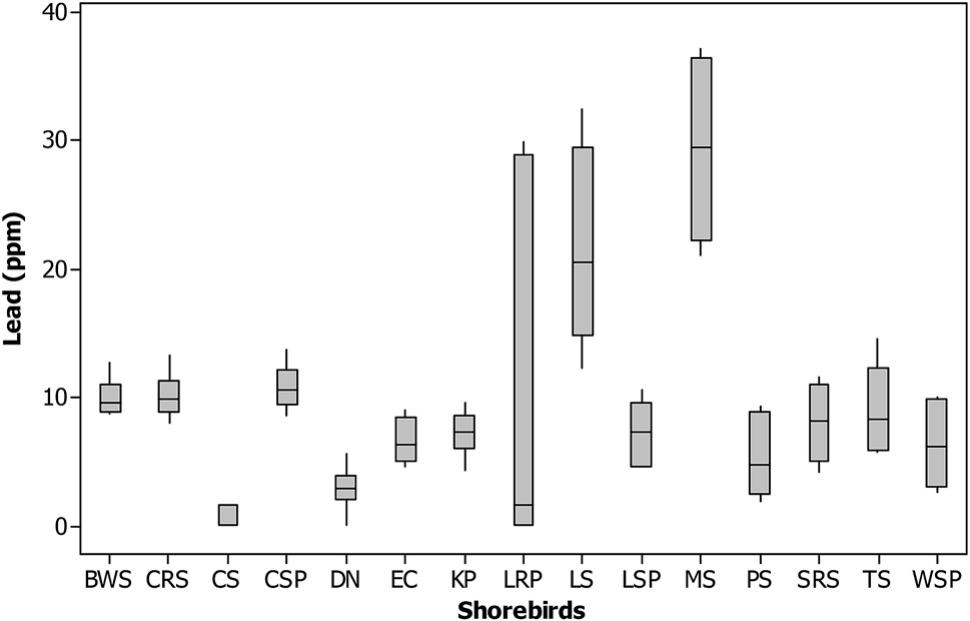
Box plot showing the concentration of lead (ppm) recorded in different shorebird species at Point Calimere Wildlie Sanctuary, Kodikkarai and Pichavaram Mangrove Forest, Pichavaram, east coast of Tamil Nadu, India.

**Figure 7:**
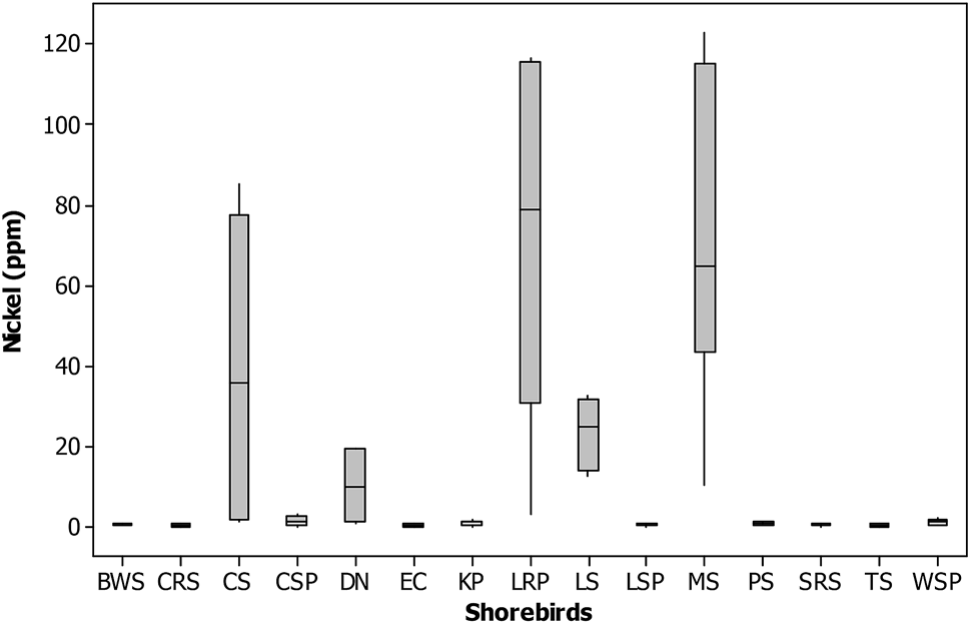
Box plot showing the concentration of Nickel (ppm) recorded in different shorebird species at Point Calimere Wildlie Sanctuary, Kodikkarai and Pichavaram Mangrove Forest, Pichavaram, east coast of Tamil Nadu, India.

**Figure 8:**
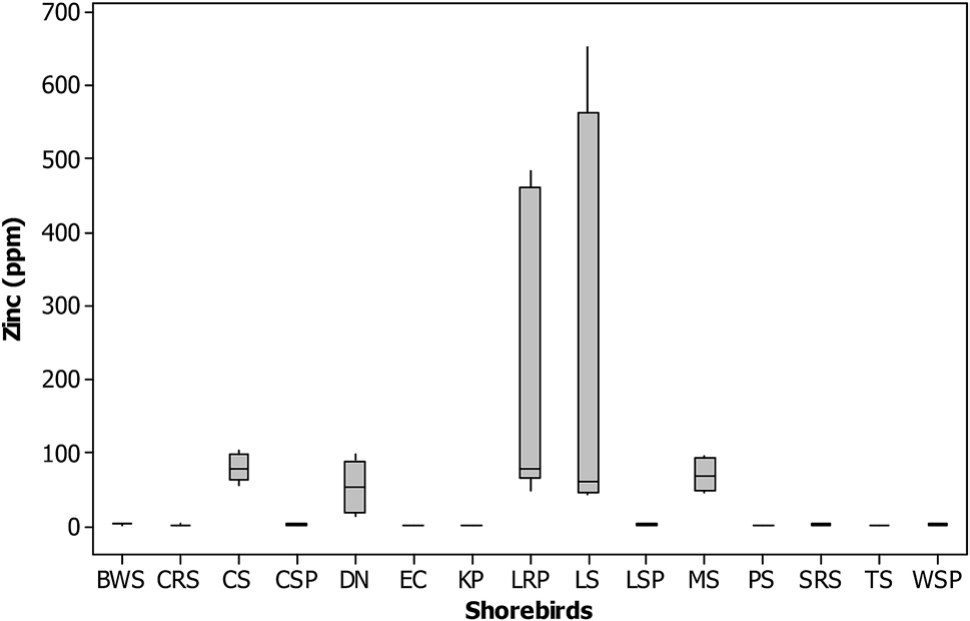
Box plot showing the concentration of Zinc (ppm) recorded in different shorebird species at Point Calimere Wildlie Sanctuary, Kodikkarai and Pichavaram Mangrove Forest, Pichavaram, east coast of Tamil Nadu, India.

The Zinc (Zn) was highest in feathers of Dunlin (90.8 ± 4.29), Little stint (419.09 ± 178.1) and Marsh sandpiper (91.9 ± 1.42), Common Sandpiper (95.6 ± 4.08) and Little ringed plover (328.4 ± 140.21 ppm) comparatively other shorebirds studied (Tables 1 and 2 and Figs. 2, 3, 4, 5, 6, 7, 8). Generally, these shorebirds are using CAF migratory routes for traveling their breeding and non-breeding grounds during their migration^7,54^, which could also facilitate the more significant accumulation of metals in these shorebirds. These shorebirds mainly feed on benthic invertebrates such as molluscs, gastropods, polychaetes, shrimp forms, amphipods^2,55–59^, these prey items were relatively higher in both the wetlands during migratory season^41,52^. Several studies stated that zinc one of the highest concentrations of metals found at benthic invertebrates of coastal mudflats^60–62^. Zn's sources in both the wetlands are through the saltpan industries, construction activities, national highways, reliable waste burning, aquaculture industries and agricultural practices, etc. The zinc is a greater water and sediment level and may be found in higher concentrations to result in avian poisonings (unpublished data). Nevertheless, beyond the standard level, the Zn might affect the biotic community, 16 mg of Zn by oral exposure for 2-weeks leads 50% mortality in cockatiels and even 2 mg of Zn/week showed toxic in some birds^63^. Zn is one of the lethal poisonings when it is exceeded in ducks^64^ and a Nicobar pigeon^65^.

Concerning the Pb, the Black-winged stilt (9.6 ± 0.42), Common Sandpiper (9.6 ± 0.26), Curlew sandpiper (9.4 ± 0.57), Eurasian curlew (6.9 ± 1.09), Lesser sand plover (9.5 ± 0.52), Temminck’s stint (6.1 ± 0.34), Kentish plover (8.3 ± 0.18), Spotted redshank (10.6 ± 0.88) and Wood sandpiper (9.5 ± 0.12 ppm) showed a greater concentration of Pb when compared to the other species of shorebirds studied from both the wetlands (Tables 1, 2 and Figs. 2–8). These species' variations might be due to their unique physiology, selection and intake quantity of various diets, and their flyway strategies. Among the nine shorebird species, the lesser sand plover is used EAAF migratory routes^66^, and the remaining eight shorebirds are used CAF routes^7,67^ for traveling their wintering and non-wintering grounds during their migration. Besides, contamination occurs mainly through sewage disposal, burning of fuels and solid wastes, making paints, transports, refining, leather industry and oil spills, etc^68,69^. A study reported that Pb is distributed in the environment through agricultural practices such as fertilizers and pesticides by the farmer communities^23^. These shorebirds mainly feed on benthic invertebrates such as crustaceans, chironomid larvae and midges, polychaete, gastropod molluscs, bivalve molluscs, amphipods and decapod crustaceans, insects, arachnids and pisces^70–72^ , which exist abundantly in both the wetlands^41,52^. Studies found that the Pb more significant in mud dwelling organisms in coastal mudflats since they are feed on detritus^73^. It could be a reason that these shorebirds are showing the moderately higher amount of Pb, since they are also consuming these detritus feeding organisms. In addition to that, these shorebird species using various stopover sites during migration before reaching these sites could also facilitate Pb accumulation. The effect of Pb level in the feathers (4lg/g) is on the weakening of thermoregulation, locomotion, weakening the survival of gull nestlings, and influencing the recognition of siblings^46^. Another study suggested that the excess amount of Pb concentration disturbs protein signaling (protein c kinase), particularly memory storage^74^. Memory storage could affect the migration of shorebirds seasonally. Nevertheless, leading a biota is an indicator of metal pollution and anthropogenic activities contribute to it^75^.

Concerning Hg, the Kentish plover, Painted stork, Spotted redshank, Wood sandpiper, Eurasian curlew and Lesser sand plover have a higher level of Mercury (Hg) than the feathers of other species shorebirds evaluated in both the wetlands. These shorebirds are used CAF migratory routes for their migration except Lesser sand plover, which is used EAAF migratory routes for migration. These shorebirds predominantly feed on benthic invertebrates during their migration in the breeding and non-breeding grounds annually^70,76^. The Kentish plover (8.3 ± 1.03), Painted stork (8.6 ± 0.60) , Spotted redshank (7.8 ± 0.55), Wood sandpiper (7.7 ± 0.82), Eurasian curlew (7.7 ± 0.46) and Lesser sand plover and Lesser sand plover (5.8 ± 0.79 ppm). (Tables 1, 2). The Painted stork showed a greater level of Hg among the shorebird species it might have due to their diets and they are predominantly feeding on locally available diets, mainly fishes because studies reported that the greater level of Hg found in the various species of fishes at PMF^77^. A higher level of Hg found in other shorebirds might be also due to their diet preference and also perhaps uptakes of Hg from various stopover sites in their migratory flyway during their migration. Most of the consumers/predators in the coastal ecosystem, such as shorebirds, expose more mercury because they feed on detrital benthic organisms, which can elevate the level of mercury^78,79^. The benthic or mud dwelling organism are exposed more to metals, including mercury because most of the benthic organisms are filter feeders and these benthic organisms are feed on detritus, which can drive elevated accumulation of metals in the benthic organisms. The metals are directly settled into the benthos by their food in sediment and water^80^. Another study explained that the shorebirds' more possibility of ingestion of sediments and their prey items and the metals, including mercury, are higher in sediment^81^. The present study showed that Hg's level was more significant than 5 ppm except for Common sandpiper, Dunlin and Marsh sandpipers compared to the other 12 species of shorebirds examined. The rising of Hg concentration in birds could distress their behavior, physiology and breeding success^82^. A study reported that if the Hg, more than 15 ppm, can have adverse effects in predatory birds^83^.

The Common Sandpiper and Little-ringed plover showed a higher level of Nickel (Ni) than the other metals found in their feathers. These two are migrant species and they are using CAF migratory routes during their migration for traveling their breeding and wintering grounds. The Common Sandpiper is forage on amphipod (46.9%), the polychaetes (39.0%) and the decapod (6.3%)^84^, polychaetes, decapods algae, seeds, worms, spiders, fish, frogs and tadpoles^58^. The Little ringed plover feeds on polychaetes, crustaceans and insects^70^. The nickel concentration in Common sandpiper and Little-ringed plover was (70.9 ± 9.75) (114.8 ± 1.18 ppm), respectively (Tables 1, 2). The current study results revealed a comparatively more incredible amount of Ni in Little egret feathers^85,86^, suggesting that its levels were lower in them than that of the current study. Sources of nickel in the shorebirds' feathers might be through their dietary sources because a study reported that the nickel is exposing in a benthic organism, including molluscs and other benthic organisms^87^. It might have been a reason that these species showed a higher amount of nickel in their feathers. Besides, the nickel was found at molluscs, crab, and polychaetes in India's coastal habitat, which are predominant prey items for shorebirds^88^. Nickel is not an essential trace element in organisms, but it can have adverse health effects at high levels. However, no sufficient evidence is available on the impact of nickel in avian species, but in animals, the level of Ni is exceeded, which will affect various cellular functions. A study reported that the excess accumulation of Ni causes pigmentation of feathers in birds and is excreted through feathers by moulting^89^.

The little stint is the only species that showed the highest amount of Cobalt (Co) in their feather (128.7 ± 54.72 ppm). However, the other species such as Dunlin (30.8 ± 16.52 ppm) and Marsh sandpiper (53.08 ± 0.94 ppm) also showed relatively higher amount of cobalt concentration when compared to the other shorebird species studied (Table 1, 2), these three species are migrant category, and they are using CAF routes for their migration for both the wintering and breeding grounds. These species also feed on benthic invertebrates, including foraminiferas, polychaetes, chironomids, crustaceans, molluscs, and larval forms of insects and insects^56,58^. Studies stated that benthic invertebrates, foraminiferas and molluscan shells have a reasonably greater level of Co in their body since their feed on detritus, including litter, debris, organic and inorganic wastes^90,91^. Conversely, the benthic prey items predominantly used by the shorebirds are densely available during migration seasons^41,52^; this could also be another reason for Co's accumulation in these shorebird species. Co is also an essential element critical for the metabolic process but can cause harmful effects when its level is extreme. Besides, cobalt disturbs certain enzymes and might have affected the birds' physiology and movement^92^.

Indeed, Marsh sandpiper had the maximum concentration of Chromium (Cr) (114.7 ± 5.39 ppm) in their feathers than the other shorebirds studied. The chromium level in the feathers of the Marsh sandpiper was lesser than the earlier reports^86^ and relatively more Cr was also reported (Burger et al. 2015) compared to the current results. The Marsh Sandpiper is forage on benthic organisms, including molluscs, polychaetes and crustaceans^58^. For instance, ample Cr is in sediment and benthic organisms^93^. Cr occurs naturally in the aquatic environment by burning organic and inorganic materials, including oil and coal, chromium steel factories, production of manures, and metal plates production in tanneries.

Nevertheless, through various human activities, it’s released into the environment through sewage and fertilisers^94^. In addition to that, the Cr is rich in coastal sediments through the sources of domestic and industrial effluents^95^. Since the Marsh sandpiper is fed on benthic invertebrates by probing methods, the sediments also enter the bird and their prey items. It is also another reason the accumulation of Cr in the feathers of the shorebird species. Besides, the Marsh sandpiper is using CAF migratory route for their wintering and breeding grounds, perhaps along the stopover sites in CAF routes could also facilitate the accumulation of the Cr in their feathers.

Nevertheless, the presence of Cr at > 4 ppm level is alarming. However, the present study shows that great Cr in shorebirds' feathers above the average (2.8 lg/g) might be harmful to them^96^. However, extensive studies are required on Cr and its impact on birds' physiology, especially reproductive toxicology, which could reveal some clues, particularly for wildlife management.

Among the shorebird species studied, Dunlin and Marsh sandpiper had a higher amount of Copper (Cu) (87.2 ± 10.62) and (32.3 ± 6.69 ppm), respectively, than the other shorebirds evaluated from both the wetlands (Tables 2,3). The presence of Cu in the environment is through various industries, factories, agricultural practices, and waste dumping. However, most aquatic habitats are receiving the Cu from agricultural practices, precipitation through the atmosphere and industrial discharges. The greater level of Cu found in the shorebirds might be through their food and feeding habits since they are feed on benthic invertebrates such as molluscs, gastropods, polychaetes, shrimp forms, amphipods in the breeding and wintering grounds during their migration^55,56^. These shorebirds are using CAF migratory routes during their migration for traveling their breeding and non-breeding grounds^7,67^. Besides, the Cu is also plenty in benthic invertebrates and sediments^91^, facilitating the Cu in the shorebirds studied. In addition to that, since these species are a migrant category which is traveling thousands of kilometers annually and they are using several stops over sites to reach their breeding and non-breeding grounds along with CAF migratory routes, which is also taking into account concerning the accumulation of Cu in the feathers of shorebirds studied. However, copper is an essential and vital trace nutrient for all known living organisms. Its role is significant in cellular mechanisms and protein functions^97^. A greater level of Cu could disturb the functions of the kidney and damage the reproductive system^98^ .

Another vital metal is Arsenic (As), which is not evaluated in the current study, but studies were reported arsenic was in shorebirds such as Red knows, Sanderling and Semipalmated sandpiper (446 ± 42 ppm), (311 ± 64 ppm) and (842 ± 101 ppm) respectively from Delaware Bay, New Jersy^88^. Compared with a previous study from 1995, the results showed that the linear trend had been observed concerning the nickel and other metals. However, a significant report by Bird Conservation International revealed that arsenic could be an adverse effect on shorebirds, reported from Pulicate lake, India during 2004^99^.

The inter-correlational analysis showed that the mercury was not positively correlated with the other metals. Studied in both the wetlands, which shows that the mercury is a unique and their deposition in the living organism not depends on the level of other metals, how the other metals have positive with certain metals which shows that these metals have interrelationships in their availability, distribution and accumulation on the living organisms (Table 3). The current study found that the level of metals showed significant variations among the shorebird species studied. Nevertheless, the variations among the shorebird species can be due to their feeding mechanism, including the type, sizes and selection of the prey species, mode of feeding mechanisms, amount of time and prey consumed in their foraging grounds, quality of the habitats etc. A study revealed that the selection of prey and feeding mechanism could facilitate higher metal concentration levels in birds^100^. The variation of metals among the shorebirds might be due to their different migratory strategy i.e., CAF and EAAF migratory routes. The migratory shorebirds are using various stopover sites to reach their wintering and breeding grounds annually. Therefore, the accumulation of metal in the shorebirds from both the wintering wetlands studied and it might be consumed from other stopover sites except resident migratory shorebirds. Nevertheless, the level of metals in the resident shorebird, i.e., Painted stork, might be influenced by these wetlands.

### Management implication

- The current study revealed that the stopover sites of Central Asian Flyway (CAF) and East Asian Australasian Flyway (EAAF) migratory routes of shorebirds, especially in southern wintering grounds, are under mental stress, which could influence bird behaviour and migration.
- It is the right time to evaluate the wetland habitats, especially IBA and Ramsar sites, to understand their current status to protect the habitat and turn the migratory shorebirds.
- Growth and development of various dimensions to meet the growing needs of human beings' growing needs and services affect resources' sustainability. Day by day, it becomes antithetic and incongruous. Hence, there is an urgent need to evolve a strategy to integrate various science streams such as avian ecology, reproductive ecology, food web ecology, aquatic ecology, toxicology, and environmental chemistry to understand any habitat's status. Otherwise, one cannot prevent species extinction under direct threat and would soon be categorized as threatened.
